# Supplementary material for: Genome-wide identification and analysis of the thiolase family in insects
Source: PeerJ. 2020 Nov 20;8:e10393. doi: 10.7717/peerj.10393 (PMC7682436; doi:10.7717/peerj.10393)

(A) Catabolic process of 3-oxoacyl-CoA thiolase

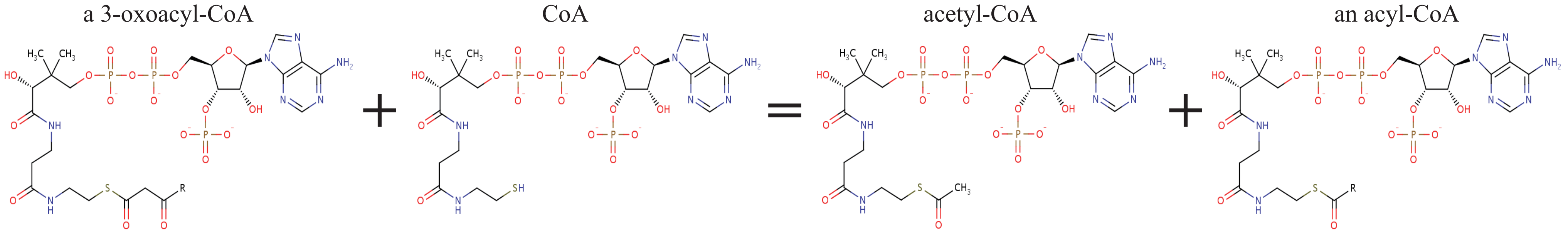

(B) Biosynthetic process of acetoacetyl-CoA thiolase

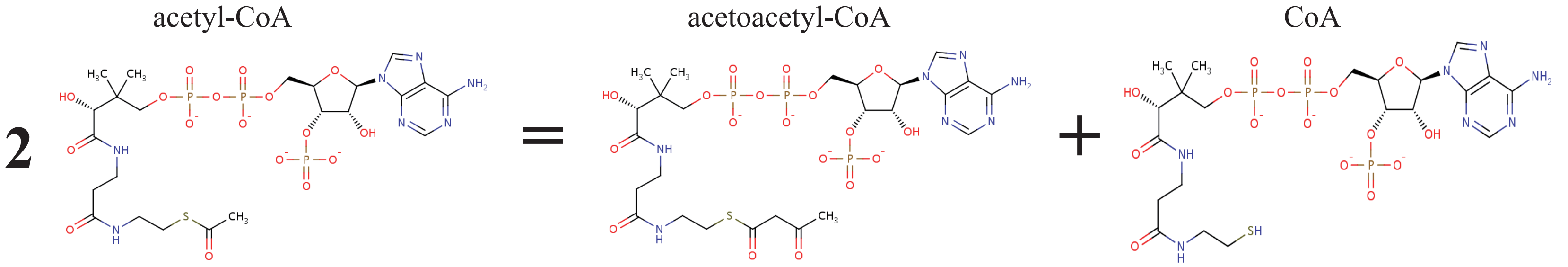

Supplement: Supplemental Information 1 — Retrieved from https://www.uniprot.org/. [file peerj-08-10393-s001.pdf]
