# Supplementary figures and images for: Genome-wide identification and analysis of the thiolase family in insects
Source: PeerJ. 2020 Nov 20;8:e10393. doi: 10.7717/peerj.10393 (PMC7682436; doi:10.7717/peerj.10393)

(A) Gene structure analysis  
■ Exon (ex.)    — Intron (in.)

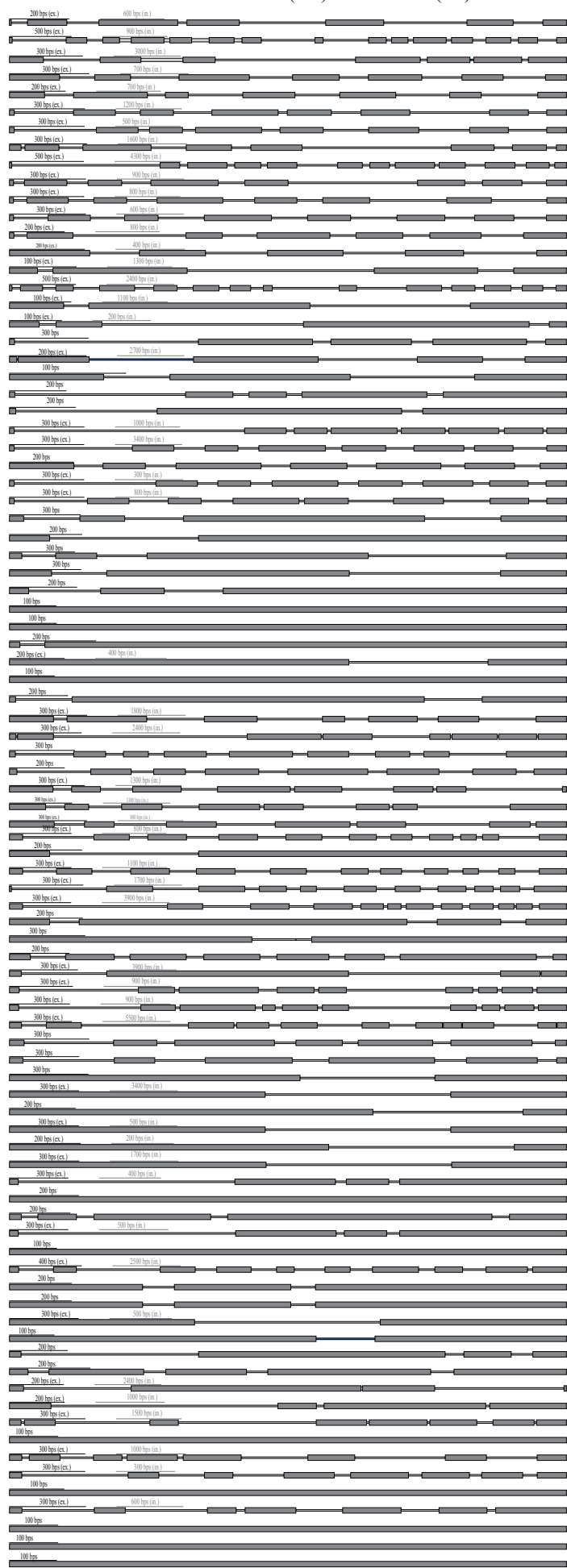

(B) Subcellular localization  
Mit Cyt Pox

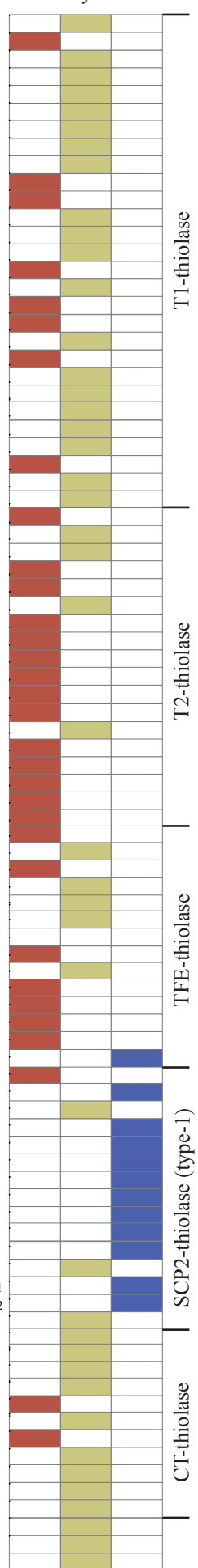

Supplement: Supplemental Information 2 — (A) Exon/intron structure analysis. (B) Subcelluar localization analysis. Mit: mitochondrion; Cyt: cytosol; Pox: peroxisome. [file peerj-08-10393-s002.pdf]

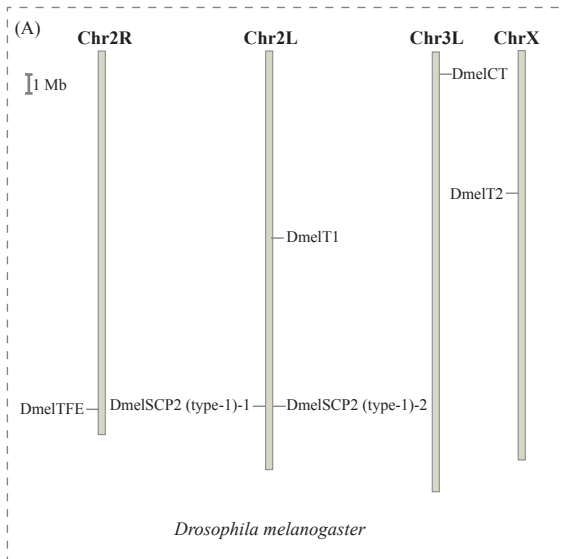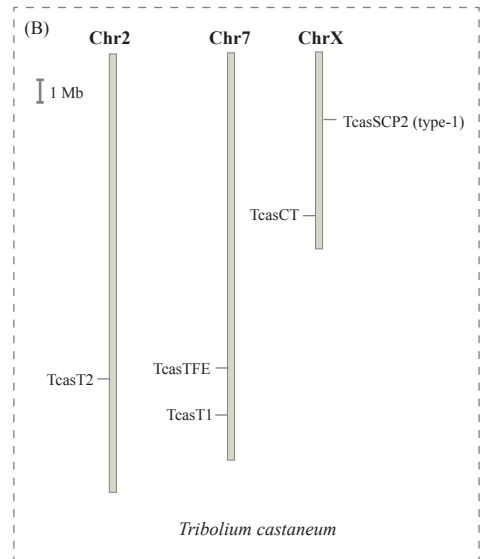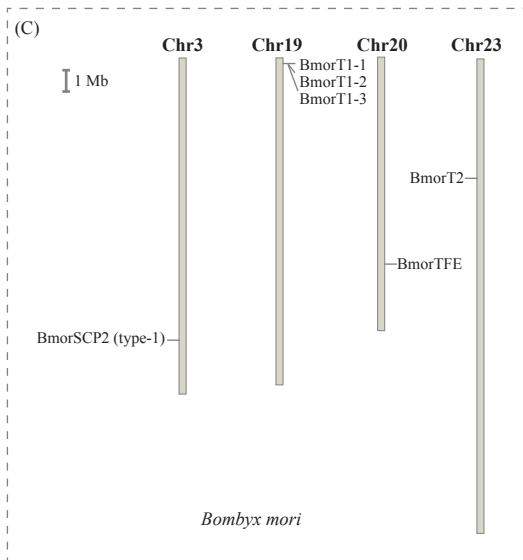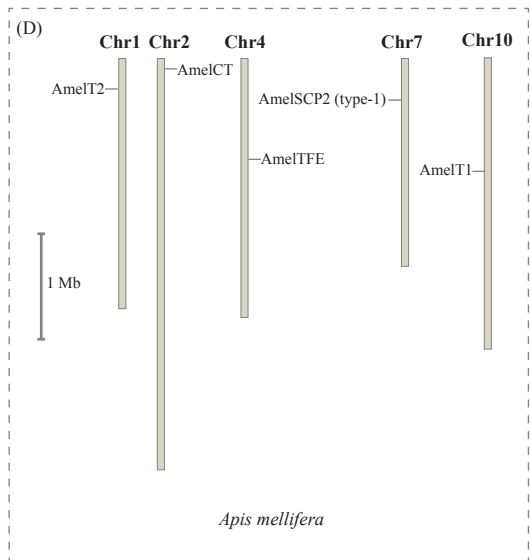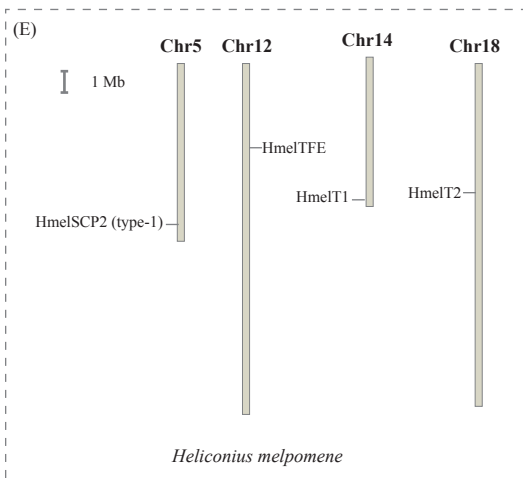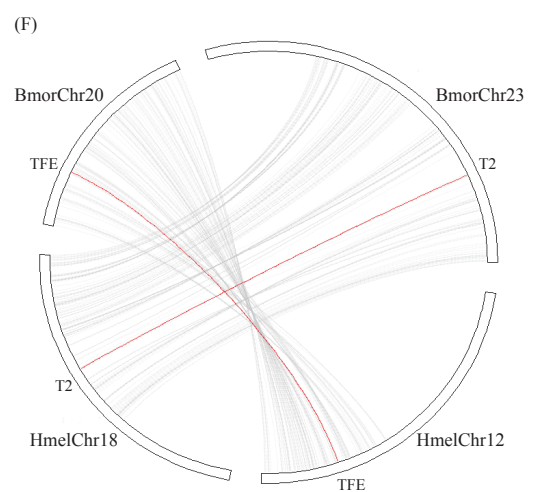

Supplement: Supplemental Information 3 — (A) to (E) Chromosomal distribution of the thiolase genes was presented for D. melanogaster, T. castaneum, B. mori, A. mellifera, H. melpomene, respectively. Chr: chromosome.(F) Synteny events of the thiolase genes betweenH. melpomene and B. mori. Gray lines indicate all synteny blocks for the 4 chromosomes and red lines indicate synteny relationships of the thiolase genes. [file peerj-08-10393-s003.pdf]

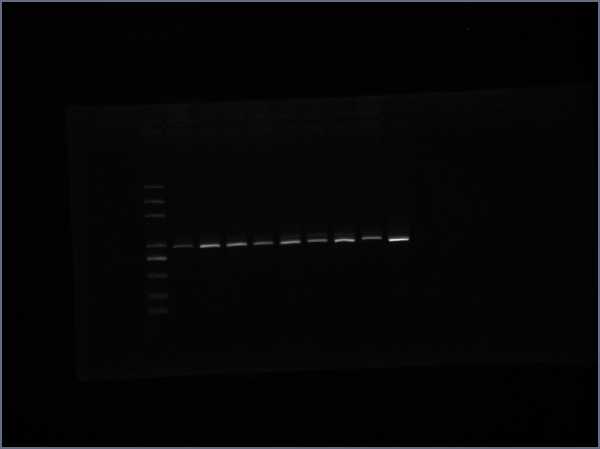

Supplement: Supplemental Information 9 [file peerj-08-10393-s009.zip › Fig.6B_uncropped blots/BmorRpL3.jpg]

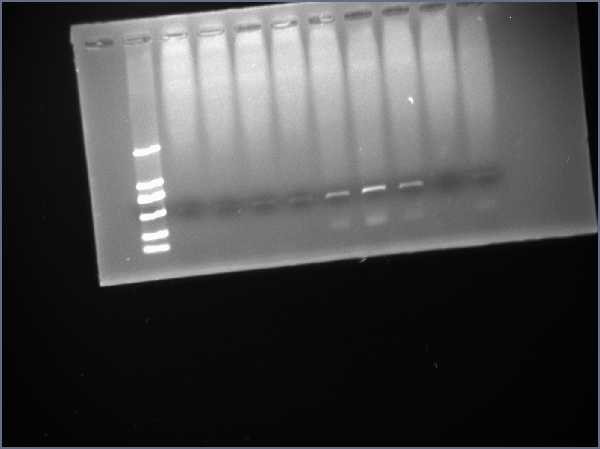

Supplement: Supplemental Information 9 [file peerj-08-10393-s009.zip › Fig.6B_uncropped blots/BmorSCP2.jpg]

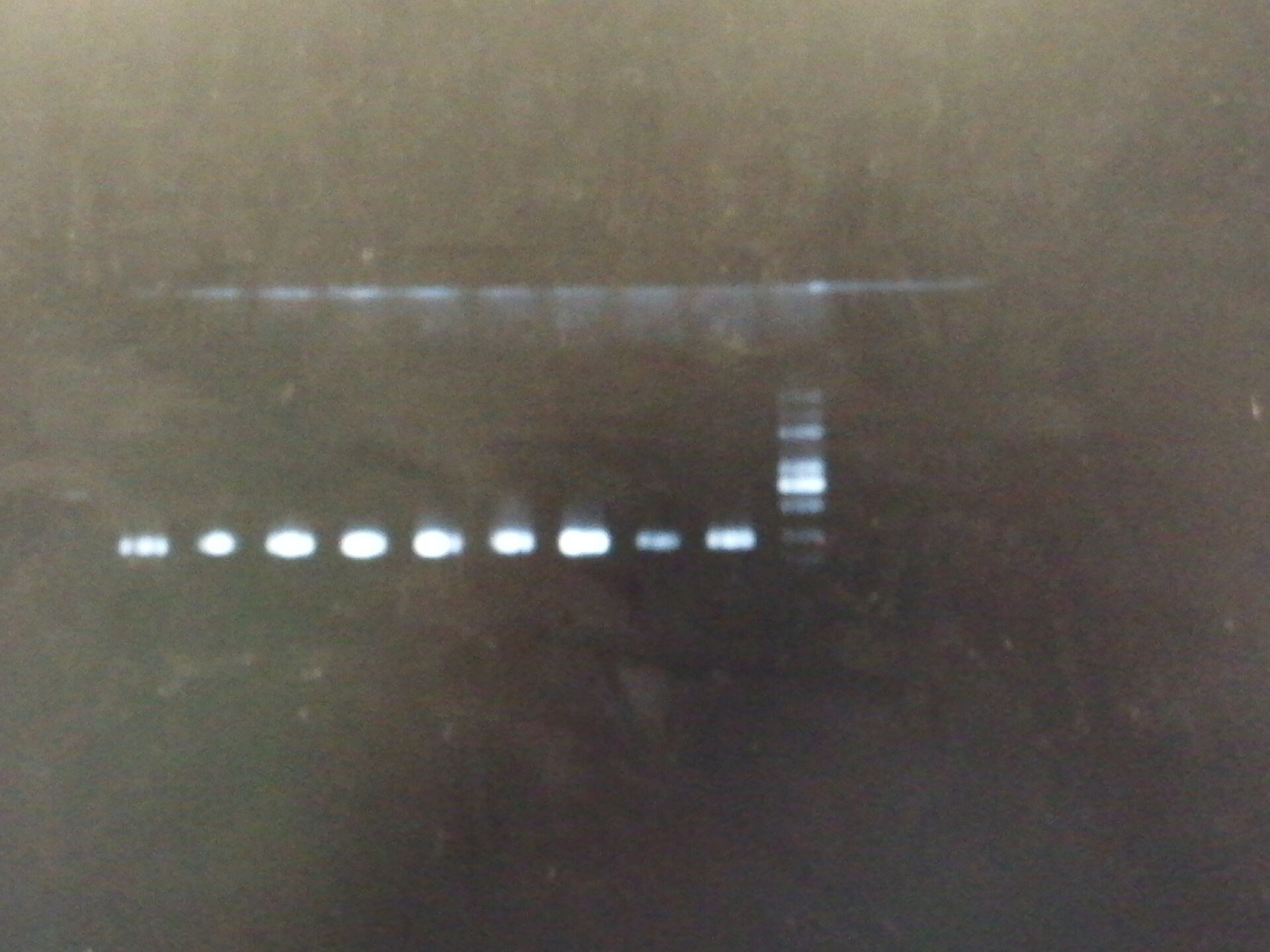

Supplement: Supplemental Information 9 [file peerj-08-10393-s009.zip › Fig.6B_uncropped blots/BmorT1-1.jpg]

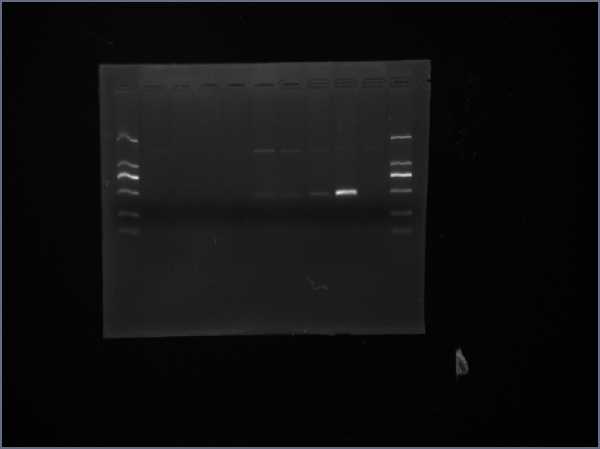

Supplement: Supplemental Information 9 [file peerj-08-10393-s009.zip › Fig.6B_uncropped blots/BmorT1-2.jpg]

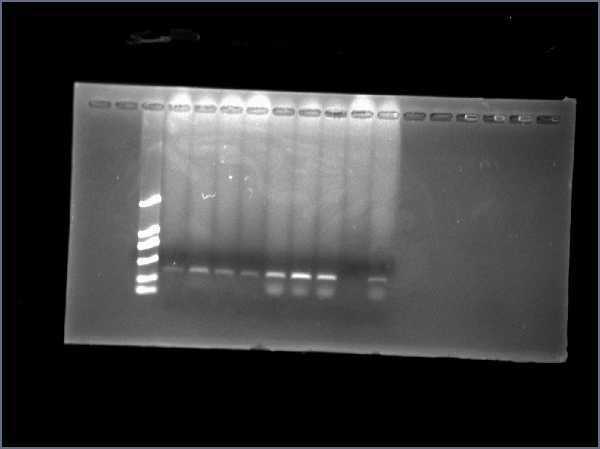

Supplement: Supplemental Information 9 [file peerj-08-10393-s009.zip › Fig.6B_uncropped blots/BmorT2.jpg]

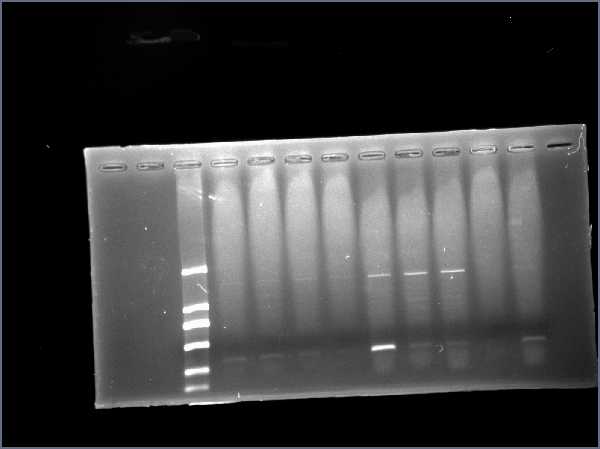

Supplement: Supplemental Information 9 [file peerj-08-10393-s009.zip › Fig.6B_uncropped blots/BmorTFE.jpg]

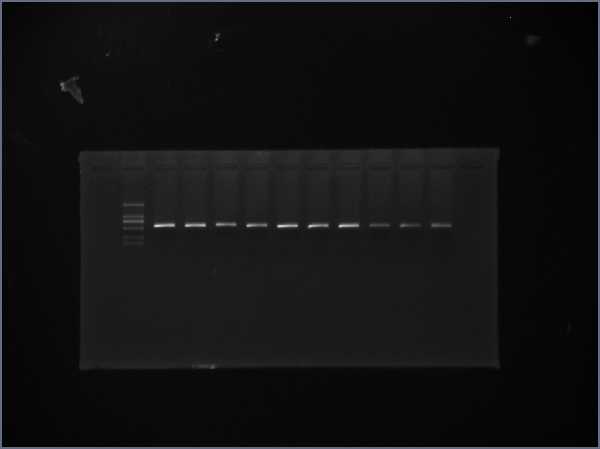

Supplement: Supplemental Information 9 [file peerj-08-10393-s009.zip › Fig.6C_uncropped blots/BmorSCP2.jpg]

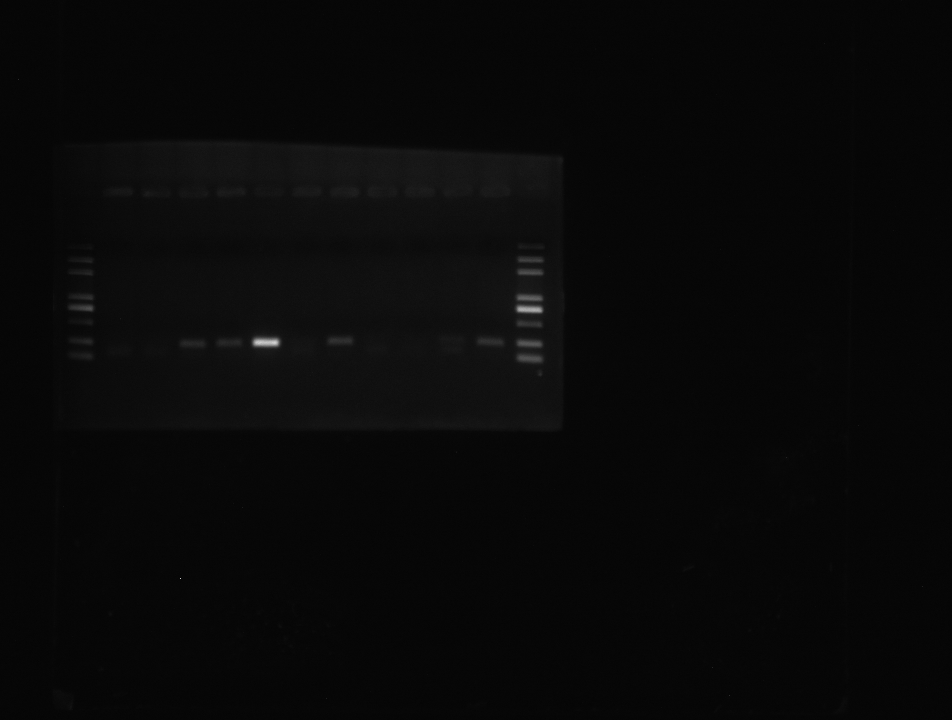

Supplement: Supplemental Information 9 [file peerj-08-10393-s009.zip › Fig.6C_uncropped blots/BmorT1-1.Tif]

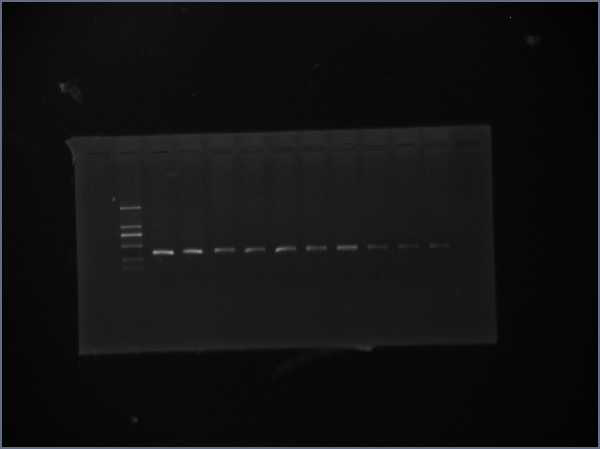

Supplement: Supplemental Information 9 [file peerj-08-10393-s009.zip › Fig.6C_uncropped blots/BmorT2.jpg]

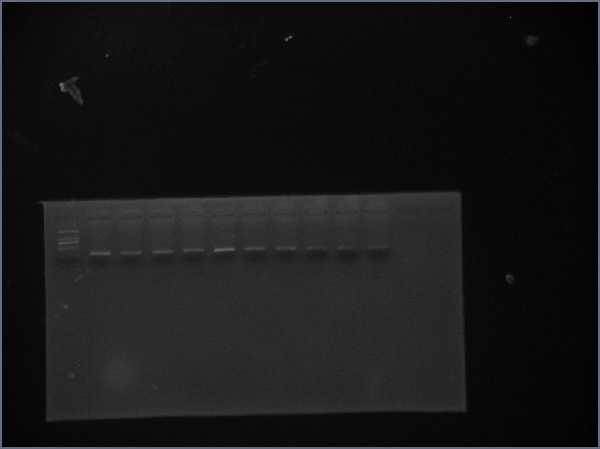

Supplement: Supplemental Information 9 [file peerj-08-10393-s009.zip › Fig.6C_uncropped blots/BmorTFE.jpg]

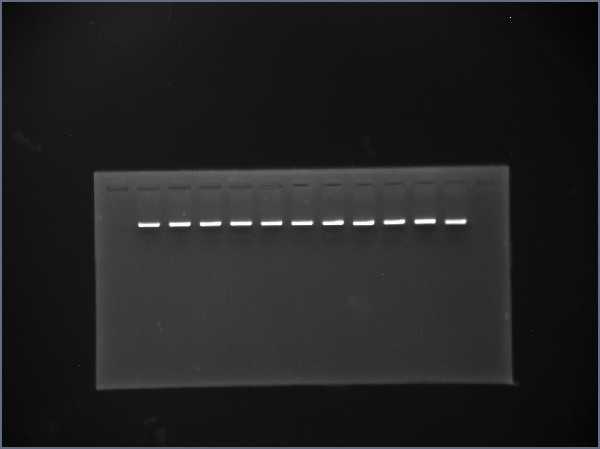

Supplement: Supplemental Information 9 [file peerj-08-10393-s009.zip › Fig.6C_uncropped blots/BmRpL3.jpg]
