# Supplementary material for: Genome-wide identification and analysis of the thiolase family in insects
Source: PeerJ. 2020 Nov 20;8:e10393. doi: 10.7717/peerj.10393 (PMC7682436; doi:10.7717/peerj.10393)
Supplement: Supplemental Information 4 [file peerj-08-10393-s004.pdf]

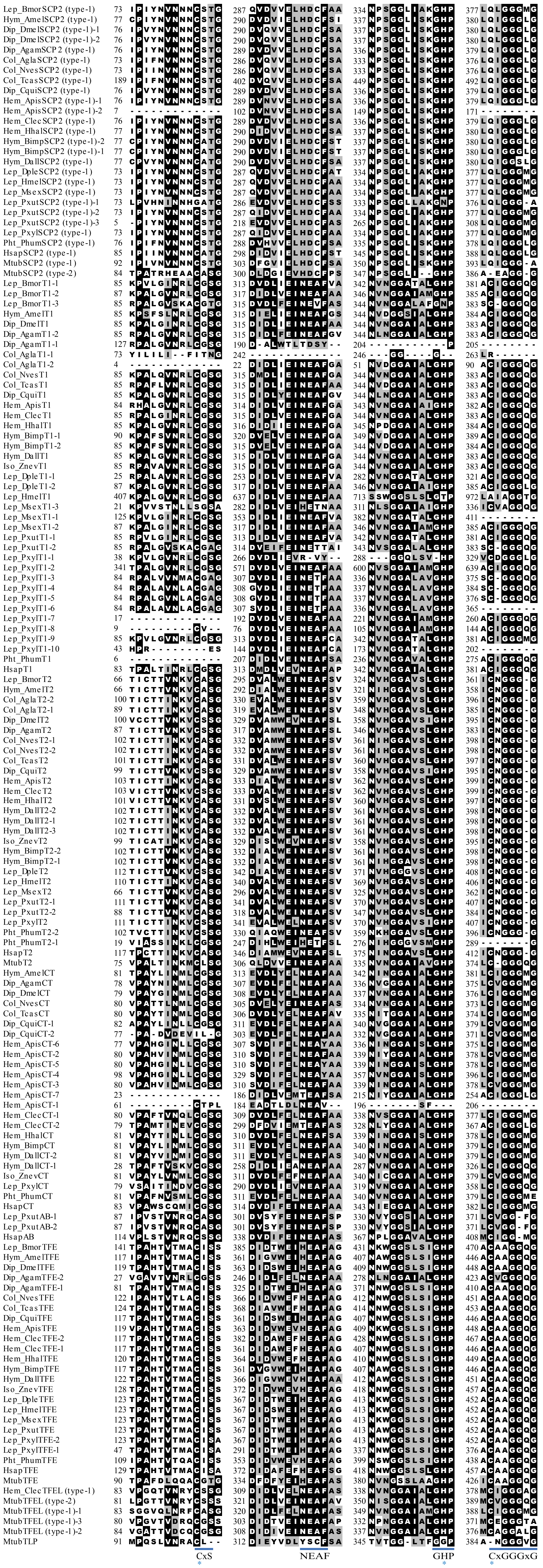
CxS
NEAF
GHP
CxGGGxG
