# Supplementary material for: Genome-wide identification and analysis of the thiolase family in insects
Source: PeerJ. 2020 Nov 20;8:e10393. doi: 10.7717/peerj.10393 (PMC7682436; doi:10.7717/peerj.10393)
Supplement: Supplemental Information 6 [file peerj-08-10393-s006.docx]

**Table S2 Primer sequences used for the RT-PCR experiment**

| **Gene name** | **Accession no.** | **Primer (5'-3')** | **Len**  **(bp)** | **Tm**  **(°C)** |
| --- | --- | --- | --- | --- |
| *BmorSCP2* | BGIBMGA009103 | F: GCTTTGGGAGGTGGTCATTT | 409 | 55 |
| *(type-1)* |  | R: TGTCAGTTGCCATCTCCATTC |  |  |
| *BmorT2* | BGIBMGA011029 | F: CCATTGACTTCCCGATAGCA | 267 | 53 |
|  |  | R: AAGCAACTCCCTTTTCTCCT |  |  |
| *BmorT1-1* | BGIBMGA012659 | F: TTACGCTCTACAATCGCAACAGAA | 203 | 55 |
|  |  | R: TTCCAGCAGTGACTAGACCTTCCTT |  |  |
| *BmorT1-2* | BGIBMGA012660 | F: GTAAGCGGAACATCAGACGG | 384 | 54 |
|  |  | R: CTGGGATTGAAGCGAAAACT |  |  |
| *BmorT1-3* | BGIBMGA012661 | F: TACAGAGGGAGTTCTTGGACAG | 384 | 53 |
|  |  | R: GGGGTTTTAGGTTTTGGGTT |  |  |
| *BmorTFE* | BGIBMGA014181 | F: GTCAAGGAAAGGTTGGCAGTC | 223 | 55 |
|  |  | R: TATTGGGTGGTGGCATCAGG |  |  |
| *BmorRpL3* | AY769270 | F: CGGTGTTGTTGGATACATTGAG | 161 | 55 |
|  |  | R: GCTCATCCTGCCATTTCTTACT |  |  |

The F and R represent forword and reverse primers, respectively. The accession number of *BmRpL3* was in GenBank. Len: length of amplificon; Tm: annealing temperature.
