# Supplementary material for: Genome-wide identification and analysis of the thiolase family in insects
Source: PeerJ. 2020 Nov 20;8:e10393. doi: 10.7717/peerj.10393 (PMC7682436; doi:10.7717/peerj.10393)
Supplement: Supplemental Information 7 [file peerj-08-10393-s007.docx]

Table S3 The root-mean-square distance (RMSD) matrix of the thiolases estimated by Swiss-PdbViewer

|  | BmorT1-1 | DmelCT | PxutAB-1 | BmorTFE | BmorSCP2 (type-1) |
| --- | --- | --- | --- | --- | --- |
| BmorT2 | 0.91 | 0.89 | 0.91 | 1.16 | 1.37 |
| BmorT1-1 |  | 0.25 | 0.91 | 1.19 | 2.01 |
| DmelCT |  |  | 0.89 | 1.16 | 1.95 |
| PxutAB-1 |  |  |  | 1.12 | 1.49 |
| BmorTFE |  |  |  |  | 2.03 |
